# The effects of listening to music in sports activities on psychological resilience, physical strength performance and motivation in terms of mental health

**DOI:** 10.3389/fpsyg.2025.1644517

**Published:** 2025-10-13

**Authors:** Sinem Becan, Nazım Serkan Burgul, Nazan Cenkova, Yasemin Sorakın, Güner Konedralı, Şeyma Elif Şimşek, Doğukan Raif Tuğcu

**Affiliations:** ^1^Faculty of Sports Sciences, Near East University, Mersin, Türkiye; ^2^Atatürk Teacher Training Academy, Mersin, Türkiye

**Keywords:** the effects of listening to music in sports activities, psychological resilience, physical strength performance, motivation, mental health

## Abstract

This research investigates the effects of listening to music on psychological resilience, physical strength performance, and motivation in sports activities, emphasizing the mental health of active athletes participating in international competitions in the Turkish Republic of Northern Cyprus (TRNC). In this study, a quantitative survey model was used. The study’s sample group consists of 344 athletes (169 female athletes and 175 male) from TRNC who participate in international competitions. Data were collected using a “Personal Information Form,” and the “Impact of Music in Sportive Activities Scale (IMSAS),” consisting of 18 items under the three sub-dimensions, developed by Karayol and Turhan (2020). The data were analyzed quantitatively using SPSS 26.0. Independent samples t-test and one-way ANOVA were used for comparisons between groups of participants based on different independent variables. In cases where significant results were obtained from ANOVA, Bonferroni tests were used for *post hoc* analysis. In this study, the relationship between the participants’ gender, type of sport, years of sport experience, and the effects of listening to music in training on their psychological resilience, physical strength performance and motivation were examined. A significant difference was found in the variables of gender and years of sport experience depending on the participants’ listening to music before, during, and after the activity. According to the gender variable, significant differences were found in the physical strength and performance and psychological resilience sub-dimensions during both the pre-activity and during-activity phases. In the post-activity phase, significant differences were observed across all three sub-dimensions: motivation, physical strength and performance, and psychological resilience. In terms of years of sports experience, significant differences were identified in all three sub-dimensions during the pre-activity phase. During the activity, significant differences were found in the motivation and physical strength and performance sub-dimensions, while in the post-activity phase, a significant difference was observed only in the psychological resilience sub-dimension. There were no statistically significant differences across the three sub-dimensions— (motivation, physical strength and performance, and psychological resilience) based on sport type in relation to whether participants listened to music before, during, or after the activity.

## 1 Introduction

It is possible to define sports not only as an activity but as a part of the triple bond established by a person with nature, culture and personality, as a lifestyle that takes place in the natural flow of life and supports physical, emotional and social development, shapes existence and social relations (Filiz, 2002). Physical training and exercise exert a multifaceted positive influence on individuals’ physiological, psychological, and biological well-being, with improvements in overall performance being a key outcome (Ekiz and Atasoy, 2021). The beneficial relationship between physical activity and health is mediated through enhanced muscular strength, endurance, cardiovascular capacity, posture, and flexibility, all of which contribute to the development of physical fitness and functional capacity (Sener et al., 2024). Furthermore, regular physical training has been shown to reduce stress-related hormones such as cortisol, thereby promoting psychological well-being, mood enhancement, and a heightened sense of relaxation and stability among athletes (Küçük and Durmuşoğlu, 2022).

In recent years, the integration of musical memory into sports practices has emerged as an innovative approach aimed at enhancing athletic performance and promoting physical relaxation (Erdal, 2005). Music, fundamentally defined as the perception, analysis, and transformation of sounds into vocal expressions (Say, 1998), has long influenced individuals and societies across various domains of life (Ekiz and Atasoy, 2021). Beyond its auditory function, music embodies a lifestyle that encompasses the human psyche, facilitates emotional release, stimulates physical activation, revives past memories, and provides an immersive experience that transcends the immediate environment (Akkuş, 2007). This multidimensional influence positions music as both a therapeutic and recreational medium, capable of modulating emotional and cognitive states throughout sports participation (Özen, 2022). Moreover, its application as a psychological and mood-regulating strategy among athletes has been well-documented, reinforcing its functional relevance in sports psychology (Stevens and Lane, 2001).

Building upon the established psychological and physiological benefits of music in sports contexts, recent scientific discussions have focused on its potential to sustain motivation and endurance during high-intensity, long-duration training sessions (Stevens and Lane, 2001). Music serves not only as a regulatory tool for mood and emotional well-being but also as an instrument for monitoring mental health and enhancing athletes’ psychological self-regulation. Specifically, competitive athletes have demonstrated the ability to manage stress and performance anxiety more effectively through the strategic use of music (Stevens and Lane, 2001). Complementing these findings, listening to various musical genres during the preparatory phase of competition has been employed as a performance-enhancing strategy, optimizing focus and arousal levels essential for peak athletic execution (Üngür, 2021).

The concept of mental health is inherently multifaceted and resists a singular, universally accepted definition, primarily because it reflects subjective experiences that differ across individuals (Dereceli et al., 2023). In this context, the therapeutic potential of sport has gained increasing scientific recognition, with numerous studies highlighting its positive contributions to psychological well-being (Kurt et al., n.d.). The growing body of research consistently underscores the relationship between participation in sports activities and improvements in mental health indicators (Kelly et al., 2018). Supporting this consensus, Kelly et al. (2018) demonstrated that engagement in physical activity and organized sports has a significantly beneficial impact on mental health outcomes.

Engagement in sports activities fosters feelings of happiness and well-being across all age groups, highlighting the universal psychological benefits of physical activity (Kurt et al., n.d.). However, to enhance the effectiveness and sustainability of sport-based mental health interventions, scholars argue for moving beyond universally accepted, monolithic approaches and instead embracing more nuanced and individualized perspectives (Petersen et al., 2023). A substantial body of empirical research supports a positive association between regular physical activity and mental health outcomes (Ntoumanis and Biddle, 2000). In this regard, mental health is conceptualized not as a static condition but as a dynamic state of well-being—one in which athletes are able to actualize their potential, find purpose in life, cultivate social connections, navigate everyday stressors and competitive pressures, and engage in processes of self-realization (Henriksen et al., 2011).

Music has been consistently shown to exert beneficial effects on individuals during sports activities, particularly in enhancing performance and psychological readiness. Empirical findings suggest that music listening improves athletes’ endurance and supports performance stability by promoting both psychological and physiological recovery mechanisms (HİS, 2021). Additionally, music facilitates the development of motor skills, strengthens motivation, and contributes positively to mental health—all of which are critical components for sustained athletic success (HİS, 2021).

Within the broader context of mental health in sport, participation in physical activity is believed to bolster psychological resilience, physical strength, and motivational levels—elements that are crucial for achieving optimal performance. Şahin and Güçlü (2019) define psychological resilience as the capacity to maintain mental control and emotional stability in the face of distractions during performance. However, athletic performance is inherently multifactorial and can be significantly influenced by stress, motivation, and environmental variables (Bayraktar and Kurtoğlu, 2004). Even elite athletes with substantial competitive experience are required to manage performance-related stress effectively to achieve success (Konter, 1996).

Physical strength, often defined as the maximal torque a person can exert at a specific velocity, serves as a fundamental determinant of athletic performance (McCall, 2014). Variations in strength capacity among athletes are largely attributable to individual physiological differences, emphasizing the need for personalized training strategies (Özen, 2022). Performance itself is conceptualized as the highest level of achievement an individual can attain within their respective sport, shaped by both innate ability and external preparation (İnal, 2000). As Bayraktar and Kurtoğlu (2004) argue, attaining optimal performance necessitates a structured, long-term, and goal-oriented training process that integrates both physical conditioning and psychological readiness.

Central to this performance development is motivation, which operates as a dynamic internal force that energizes individuals, fosters emotional involvement, and channels behavior toward defined objectives (Bayraktar and Kurtoğlu, 2004). Since individuals may derive motivation from diverse internal and external sources, identifying these factors is essential for nurturing both personal growth and sustained athletic success (Ersoy and Başer, 2010).

This study posits that the incorporation of music throughout all phases of sports participation can meaningfully enhance key psychological and physical parameters, including psychological resilience, physical strength, and motivational drive. These effects are especially salient in the context of mental health, where music functions as a modulatory tool supporting emotional stability and cognitive focus. In light of these considerations, the present research aims to examine the influence of music on mental health, psychological resilience, physical strength, athletic performance, and motivation within diverse sporting contexts.

## 2 Materials and methods

### 2.1 Method

In this study, to examine the effects of listening to music in sportive practices in terms of mental health, psychological resilience, physical strength performance and motivation, a relational quantitative survey design was used. Quantitative research method examines the relationship of variables counted with measurement tools and analyzed by statistical methods within the scope of subjective theories (Creswell and Creswell, 2017). The survey model is a research design that aims to determine the existence of a problem in the past or ongoing situations (Creswell and Creswell, 2017; Creswell et al., 2021).

### 2.2 Research group

The population of the study consists of 922 athletes who are actively involved in sports activities in the Turkish Republic of Northern Cyprus (TRNC) and are participating in international competitions. The study sample consists of 344 athletes (169 female athletes and 175 male athletes) chosen by purposive sampling. Purposive sampling categorizes systematically samples for research purposes (Marshall and Rossman, 2014). In purposive sampling, which is selected by the study, the sample group is selected from appropriate and voluntary participants so that the participants can easily access the study (Gravetter et al., 2009).

### 2.3 Data collection tool

The data for this study were collected using the Impact of Music in Sportive Activities Scale (IMSAS), developed by Karayol and Turhan (2020). This instrument is a 5-point Likert-type scale consisting of 18 items grouped under three sub-dimensions: Motivation (items 1–5; min: 5, max: 25), Physical Strength and Performance (items 6–11; min: 6, max: 30), and Psychological Resilience (items 12–18; min: 7, max: 35). The internal consistency of the scale, as measured by Cronbach’s Alpha, was found to be 0.885, indicating high reliability. Item-total correlations range between 0.43 and 0.57, showing acceptable levels of contribution from individual items.

The scale is applied across three distinct phases of sports activity: before, during, and after performance, allowing for a comprehensive assessment of music’s effects throughout the sporting process.

### 2.4 Research procedure

In this study, Google Forms-based “Personal Information Form” and the Impact of Music in Sportive Activities Scale (IMSAS), developed by the researcher, were sent to athletes in the study population via e-mail and WhatsApp. Feedback was received from 344 volunteer athletes. The data collection process commenced only after obtaining the necessary ethical approval from the Scientific Research and Publication Ethics Committee of Near East University (approval date: 03 March 2025; decision number: YDÜ/EB/2025/1166). Prior to completing the survey, participants were provided with an informed consent form detailing the study’s purpose, confidentiality, and voluntary nature. The survey took approximately 20 min to complete.

To ensure data quality, attention check questions were included to identify inattentive or inconsistent responses. The data collection spanned 4 weeks, during which responses were continuously monitored for completeness and accuracy; incomplete or suspicious submissions were excluded. All data were securely stored in a password-protected Google Drive account accessible only to authorized researchers. Participant anonymity was strictly maintained by separating personal identifiers from survey responses.

### 2.5 Participants

Data were collected from 364 participants for the conduct of the study. However, the data of 20 participants who filled out incorrectly or showed extreme values were removed and the study was completed with the data of 344 participants. Some demographic information of the athletes is presented in Table 1.

**Table 1:** Demographic information of the athletes.

Variable	Participants	*N*	%
Gender	Male	175	50.9
	Female	169	49.1
Sports branch	Individual sports	226	65.7
	Team sports	118	34.3
Years of experience in sports	3–5 years	86	25.0
	5–7 years	58	16.9
	7–9 years	82	23.8
	10+ years	118	34.3
	Total	344	100.0

The table displays the demographic distribution of the 344 athletes who participated in the study. Gender distribution was nearly equal, with 50.9% male and 49.1% female athletes. A majority of participants (65.7%) were engaged in individual sports, while 34.3% competed in team sports. In terms of sports experience, 34.3% of athletes reported more than 10 years of experience, while 25.0% had 3–5 years, 23.8% had 7–9 years, and 16.9% had 5–7 years. These data reflect a diverse sample in terms of both sport type and experience level.

### 2.6 Data analysis

Standardized z scores were calculated during the detection of extreme values from the answers given by the athletes to the scales. In this context, those whose z scores were not within the ±3 value range were removed from the data set. The athletes’ responses to the effect of music in sports activities were examined in the three-part sequence of sports activity: pre-practice, in-practice, and post-practice. Accordingly, the descriptive statistics of the data are given in Table 2.

**Table 2:** Descriptive statistics of the effect of music in sports activities.

Period	Variable	*N*	X_	Std. Dev.	Min	Max	Skewness	Kurtosis
Before	Psychological resilience	344	27.83	6.24	7	35	−0.877	0.292
	Physical strength performance	344	24.02	5.20	8	30	−0.842	0.165
	Motivation	344	21.06	3.94	7	25	−1.163	1.315
	Effect of music in sport applications	344	72.91	14.34	24	90	−0.868	0.345
During	Psychological resilience	344	27.43	6.59	7	35	−0.887	0.233
	Physical strength performance	344	24.08	5.31	6	30	−1.011	0.798
	Motivation	344	21.10	4.18	6	25	−1.305	1.719
	Effect of music in sport applications	344	72.62	15.09	20	90	−1.010	0.782
After	Psychological resilience	344	27.32	6.42	7	35	−0.761	0.016
	Physical strength performance	344	22.35	6.02	6	30	−0.666	−0.048
	Motivation	344	19.28	5.10	5	25	−0.812	0.097
	Effect of music in sport applications	344	68.94	16.49	18	90	−0.661	−0.052

The skewness and kurtosis coefficient was examined to examine the effect of music on athletes’ sportive practices. In this context, it is stated that it should be in the +−2 value range (George and Mallery, 2016). When the obtained data were examined, it was seen that the normality assumption was met. Therefore, parametric methods were used to analyze the data. Levene’s test was used for two independent variables, Independent Samples *t*-test was used considering the homogeneity of variance, and One-Way ANOVA was used for more than two independent variables. When significant results were obtained in ANOVA, if the variances were homogeneous, Games Howell was used if Bonferroni was not one of the Post-Hoc tests. For significant results, Cohen’s *d* was calculated for *t*-test and eta square was calculated for One-Way ANOVA. 0.20 = Small effect; 0.50 = Medium effect; 0.80 = Large effect (Cohen, 2013).

## 3 Findings

Table 3 examines the effect of music before physical training on athletes’ sports activities according to the gender.

**Table 3:** The effect of music in sports activities of athletes according to gender before physical training.

Variable	Gender	*N*	X_	Std. dev	*t* (Sd = 342)	*p* (*d*)
Psychological resilience	Male	175	26.87	6.53	−2.907	0.004* (*d = 0.31*)
	Female	169	28.81	5.78		
Physical strength performance	Male	175	23.45	5.44	−2.086	0.038* (*d = 0.22*)
	Female	169	24.61	4.88		
Motivation	Male	175	20.67	4.17	−1.874	0.062 (*d = 0.20*)
	Female	169	21.47	3.65		
Effect of music in sport applications	Male	175	70.99	14.97	−2.538	0.012* (*d = 0.27*)
	Female	169	74.89	13.41		

*p < 0.05; *d*, Cohen’s *d*.

The effect of listening to music before sports activities was analyzed according to gender of the athletes. It was determined that there was a statistically significant difference based on gender in the effect of listening to music before sportive practices (*p* < 0.05). When the source of this difference was examined, it was observed that it favored women in all dimensions except the Motivation dimension (*p* > 0.05). According to this, the effect of listening to music before sports activities on women is higher than on men.

Table 4 examines the effect of listening to music during physical training on athletes’ sports activities according to the gender.

**Table 4:** The effect of listening to music during physical training on athletes’ sports activities according to the gender.

Variable	Gender	*N*	X_	Std. dev.	*t* (Sd = 342)	*p* (*d*)
Psychological resilience	Male	175	26.57	6.81	−2.479	0.014* (*d = 0.27*)
	Female	169	28.32	6.24		
Physical strength performance	Male	175	23.46	5.63	−2.221	0.027* (*d = 0.24*)
	Female	169	24.73	4.89		
Motivation	Male	175	20.68	4.40	−1.911	0.057 (*d = 0.21*)
	Female	169	21.54	3.91		
Effect of music in sport applications	Male	175	70.71	15.84	−2.395	0.017* (*d = 0.26*)
	Female	169	74.59	14.05		

*p < 0.05.

The effect of listening to music during sports activities was analyzed according to gender of the athletes. It was determined that there was a statistically significant difference in the effect of listening to music in during physical training according to gender (*p* < 0.05). When the source of this difference was examined, it was observed that it favored women in all dimensions except the Motivation dimension (*p* > 0.05). According to this, it can be said that the effect of listening to music during sports activities on women is higher than on men.

Table 5 examines the effect of listening to music after physical training on athletes’ sports activities according to the gender.

**Table 5:** The effect of listening to music after physical training on athletes’ sports activities according to the gender.

Variable	Gender	*N*	X_	Std. dev.	*t* (Sd = 342)	*p* (*d*)
Psychological resilience	Male	175	26.18	6.60	−3.382	0.001* (*d = 0.37*)
	Female	169	28.49	6.03		
Physical strength performance	Male	175	21.49	5.99	−2.741	0.006* (*d = 0.30*)
	Female	169	23.25	5.94		
Motivation	Male	175	18.58	5.05	−2.585	0.010* (*d = 0.28*)
	Female	169	19.99	5.07		
Effect of music in sport applications	Male	175	66.25	16.43	−3.122	0.002* (*d = 0.34*)
	Female	169	71.73	16.13		

*p < 0.05.

The effect of listening to music during sports activities was analyzed according to gender of the athletes. The results show that there was a statistically significant difference based on gender (*p* < 0.05). When the source of this difference was examined, it was observed that it favored women in all dimensions. Accordingly, the effect of listening to music after sports activities on women is higher than on men.

Table 6 examines the effect of listening to music before physical training on athletes’ sports activities based on the type of sport they performed.

**Table 6:** The effect of listening to music before physical training on athletes’ sports activities based on the type of sport they perform.

Variable	Type of sport	*N*	X_	Std. dev.	*t* (Sd = 342)	*p*
Psychological resilience	Individual sport	226	27.52	6.35	−1.267	0.206
	Team sport	118	28.42	6.00		
Physical strength performance	Individual sport	226	24.00	5.23	−0.064	0.949
	Team sport	118	24.04	5.16		
Motivation	Individual sport	226	21.10	3.83	0.246	0.806
	Team sport	118	20.99	4.15		
Effect of music in sport applications	Individual sport	226	72.62	14.34	−0.506	0.613
	Team sport	118	73.45	14.38		

*p < 0.05.

The effect of listening to music before physical training on athletes’ sports activities based on the type of sport they performed was examined. The results showed that there was no statistically significant difference based on the type of sports the participating athletes did in the effect of listening to music before sports activities (*p* < 0.05). In other words, listening to music before sports activities does not impact the athletes performance according to the type of sport they do.

Table 7 examines the effect of listening to music during physical training on athletes’ sports activities based on the type of sport they performed.

**Table 7:** The effect of listening to music during physical training on athletes’ sports activities based on the type of sport they performed.

Variable	Type of sport	*N*	X_	Std. dev.	*t* (Sd = 342)	*p*
Psychological resilience	Individual sport	226	27.39	6.68	−0.159	0.874
	Team sport	118	27.51	6.44		
Physical strength performance	Individual sport	226	24.35	5.26	1.283	0.200
	Team sport	118	23.58	5.39		
Motivation	Individual sport	226	21.37	4.17	1.634	0.103
	Team sport	118	20.59	4.17		
Effect of music in sport applications	Individual sport	226	73.11	15.08	0.833	0.405
	Team sport	118	71.68	15.12		

*p < 0.05.

The effect of listening to music during physical training on athletes’ sports activities based on the type of sport they performed was examined. The results indicated that there was no statistically significant difference based on the type of sports the participating athletes did in the effect of listening to music during sports activities (*p* < 0.05). Accordingly, it can be argued that, the effect of listening to music during sports activities does not differ according to the type of sport performed.

Table 8 examines the effect of listening to music after physical training on athletes’ sports activities based on the type of sport they performed.

**Table 8:** The effect of listening to music after physical training on athletes’ sports activities based on the type of sport they performed.

Variable	Type of sport	*N*	X_	Std. dev.	*t* (Sd = 342)	*p*
Psychological resilience	Individual sport	226	27.20	6.44	−0.470	0.639
	Team sport	118	27.54	6.41		
Physical strength performance	Individual sport	226	22.24	6.13	−0.462	0.645
	Team sport	118	22.56	5.83		
Motivation	Individual sport	226	19.12	5.26	−0.765	0.445
	Team sport	118	19.57	4.79		
Effect of music in sport applications	Individual sport	226	68.57	16.76	−0.588	0.557
	Team sport	118	69.67	16.01		

*p < 0.05.

The effect of listening to music after physical training on athletes’ sports activities based on the type of sport they performed was examined. The results indicated that there was no statistically significant difference based on the type of sports the participating athletes did in the effect of listening to music before sports activities (*p* < 0.05). Thus, it can be said that the effect of listening to music after sports activities does not differ according to the type of sport performed.

The results of the examination of the differences in the effect of listening to music before sports activities based the number of years of experience in sports is given in Table 9.

**Table 9:** Effect of listening to music before physical training on athletes’ sports activities based on the number of years of experience in sports.

Variable	Years of experience	*N*	X_	Std. dev.	*F*(3–340)	*p* (*η*^2^)	Significant difference
Psychological resilience	3–5 years^a^	86	26.80	7.29	4.509	0.004* (*η*^2^ = 0.04)	d > b
	5–7 years^b^	58	25.90	6.32			
	7–9 years^c^	82	28.62	5.75			
	10+ years^d^	118	28.97	5.37			
Physical strength performance	3–5 years^a^	86	23.44	5.66	5.616	0.001* (*η*^2^ = 0.05)	c > b d > b
	5–7 years^b^	58	21.98	5.34			
	7–9 years^c^	82	24.40	5.04			
	10+ years^d^	118	25.17	4.56			
Motivation	3–5 years^a^	86	20.78	4.06	6.551	0.000* (*η*^2^ = 0.06)	c > b d > b
	5–7 years^b^	58	19.26	4.60			
	7–9 years^c^	82	21.43	3.53			
	10+ years^d^	118	21.91	3.47			
Effect of music in sport applications	3–5 years^a^	86	71.02	15.88	6.081	0.000* (*η*^2^ = 0.05)	c > b d > b
	5–7 years^b^	58	67.14	15.14			
	7–9 years^c^	82	74.45	13.02			
	10 + years^d^	118	76.04	12.65			

*p < 0.05.

The letters a, b, c, and d represent groups based on years of sports experience: (a = 3–5 years, b = 5–7 years, c = 7–9 years, d = 10 or more years).

The effect of listening to music before physical training on athletes’ sports activities based on the number of years of experience in sports was examined and a significant difference was observed in the results (*p* < 0.05). When the Bonferroni test results were examined, it was observed that those with 10 years or more experience in sports had statistically significantly higher averages in the Psychological Resilience dimension than those with 5–7 years of experience. In addition, athletes with 5–7 years of experience scored statistically lower than those with 7–9 and 10 years and more experience. Accordingly, it can be said that listening to music before the sports activities affects those with more experience in sports.

The results of the examination of the differences in the effect of listening to music during sports activities based the number of years of experience in sports is given in Table 10.

**Table 10:** Effect of listening to music during physical training on athletes’ sports activities based on the number of years of experience in sports.

Variable	Years of experience	*N*	X_	Std. dev.	*F*(3–340)	*p* (*η*^2^)	Significant difference
Psychological resilience	3–5 years^a^	86	26.50	7.34	2.473	0.062	-
	5–7 years^b^	58	26.14	6.61			
	7–9 years^c^	82	27.77	6.35			
	10+ years^d^	118	28.51	6.02			
Physical strength performance	3–5 years^a^	86	23.77	5.43	3.388	0.018* (*η*^2^ = 0.03)	d > b
	5–7 years^b^	58	22.47	5.72			
	7–9 years^c^	82	24.11	5.01			
	10+ years^d^	118	25.09	5.06			
Motivation	3–5 years^a^	86	21.03	4.22	3.110	0.027* (*η*^2^ = 0.03)	d > b
	5–7 years^b^	58	19.81	4.61			
	7–9 years^c^	82	21.04	4.03			
	10 + years^d^	118	21.83	3.92			
Effect of music in sport applications	3–5 years^a^	86	71.30	15.86	3.156	0.025* (*η*^2^ = 0.03)	d > b
	5–7 years^b^	58	68.41	15.95			
	7–9 years^c^	82	72.91	14.19			
	10+ years^d^	118	75.43	14.26			

*p < 0.05.

The effect of listening to music during physical training on athletes’ sports activities based on the number of years of experience in sports was examined and a significant difference was observed in the results (*p* < 0.05), except for in Psychological Resilience (*p* > 0.05). When the Bonferroni test was run, the difference was observed between two groups. Those with 5–7 years of experience had statistically lower scores than those with an experience of 10 years or more. Accordingly, it can be said that listening to music during the sports activities affects those with more experience in sports.

Table 11 examines the results of the examination of the differences in the effect of listening to music after sports activities based the number of years of experience in sports.

**Table 11:** Effect of listening to music after physical training on athletes’ sports activities based on the number of years of experience in sports.

Variable	Years of experience	*N*	X_	Std. dev.	*F*(3–340)	*p* (*η*^2^)	Significant difference
Psychological resilience	3–5 years^a^	86	26.59	7.23	3.385	0.018* (*η*^2^ = 0.03)	d > b
	5–7 years^b^	58	25.52	6.50			
	7–9 years^c^	82	27.62	6.14			
	10+ years^d^	118	28.52	5.73			
Physical strength performance	3–5 years^a^	86	22.08	6.63	2.596	0.052	
	5–7 years^b^	58	20.62	5.95			
	7–9 years^c^	82	22.57	5.50			
	10+ years^d^	118	23.25	5.81			
Motivation	3–5 years^a^	86	19.07	5.60	1.969	0.118	
	5–7 years^b^	58	17.98	5.03			
	7–9 years^c^	82	19.49	4.70			
	10+ years^d^	118	19.92	4.96			
Effect of music in sport applications	3–5 years^a^	86	67.74	18.75	2.993	0.031* (*η*^2^ = 0.03)	d > b
	5–7 years^b^	58	64.12	16.29			
	7–9 years^c^	82	69.68	14.96			
	10+ years^d^	118	71.68	15.39			

*p < 0.05.

The effect of listening to music after physical training on athletes’ sports activities based on the number of years of experience in sports was examined and a significant difference was observed in the results (*p* < 0.05) except for the dimensions of Physical Strength Performance and Motivation (*p* > 0.05). When the Bonferroni test results were analyzed for this variable, it was observed that those with 5–7 years of experience had statistically lower scores on the scales than those with an experience of 10 years or more. Accordingly, it can be said that listening to music after the sports activities affects those with more experience in sports.

## 4 Discussion

This study aimed to examine the effects of music listening on psychological resilience, physical performance, and motivation among athletes participating in international competitions in the Turkish Republic of Northern Cyprus (TRNC), considering variables such as gender, type of sport, and years of sports experience. The study comprehensively analyzed how music use across the three stages of athletic practice—before, during, and after training—relates to these variables.

Findings revealed that the effect of music differed significantly by gender. Before and during training, significant differences were observed in favor of female athletes across all dimensions except motivation. After training, women scored higher in all sub-dimensions. These results suggest that female athletes benefit more from music in sports settings compared to male athletes. This finding aligns with the study by Bektaş and Demir (2022), which reported significant differences favoring women in the sub-dimension of physical resilience. This conclusion was also supported by the fact that a similar study by Koç and Koç (2023) found a difference in favor of women in the physical endurance sub-dimension. Another study on basketball training found that women experienced more positive effects from music before, during, and after training compared to men (Bentouati et al., 2023). On the other hand, studies such as Ekiz and Atasoy (2021) have reported differences favoring male athletes, while others found no gender-based differences at all (Vural et al., 2019; Turhan, 2021; Turhan and Karayol, 2022). These conflicting findings may be attributed to variations in sample characteristics, sports experience, sport type, and other individual factors, as well as the influence of multiple internal and external variables inherent to athletic activities.

Regarding the type of sport, no significant differences were found in the impact of music across the three stages of athletic practice. Although direct comparisons between individual and team sports are limited in the literature, studies indicate that music listening in individual sports positively affects physical performance, motivation, and recovery (Bektaş and Demir, 2022; Vatansever et al., 2018; Vural et al., 2019; Mavi, 2012; Birnbaum et al., 2009; Çakmakçı, 2021; Kuter and Ozturk, 1997). In team sports, music has been reported to enhance group dynamics and increase motivation (Koç and Koç, 2023; Karageorghis and Priest, 2012; Terry et al., 2020; Arıkan and Akoğuz-Yazıcı, 2022). These findings suggest that music listening may have a supportive effect on mental well-being regardless of the type of sport.

In terms of sports experience, athletes with longer sports histories benefited more from music, especially before training. Koç and Koç (2023) also found that individuals with four or more years of sports experience perceived greater effects from music. In contrast, Bektaş and Demir (2022) reported that less experienced athletes benefited more from music in terms of motivation, but found no significant differences in psychological or physical resilience. Other studies argue that sports experience does not significantly affect the impact of music (Turhan, 2021; Turhan and Karayol, 2022). These differences may be due to demographic and psychosocial characteristics or individual interactions with music.

Overall, the findings suggest that music listening before, during, and after training positively influences physical performance, psychological resilience, and motivation (Bentouati et al., 2023; Penedo and Dahn, 2005; Vatansever et al., 2018; Jing and Xudong, 2008; Ooishi et al., 2017; Messaoudi et al., 2024; Miras-Moreno et al., 2025; Chen et al., 2025; Greco et al., 2024). Furthermore, music emerges as a supportive tool for mental health (Penedo and Dahn, 2005). For example, fast-tempo music may enhance performance during maximal exercise, while slow-tempo music may facilitate recovery afterward (Vatansever et al., 2018). In aerobic exercise, relaxing music can support cardiovascular function and reduce fatigue (Jing and Xudong, 2008). Ooishi et al. (2017) investigated the effects of music tempo on physiological arousal and emotional response, finding that slow-tempo music induces relaxation, while fast-tempo music elicits excitement. Accordingly, the findings of the present study are consistent with the broader literature supporting the beneficial role of music in sports contexts.

## 5 Conclusion and recommendations

As a result, this study comprehensively investigated the effects of music listening on psychological resilience, physical performance, and motivation among athletes participating in international competitions across three stages of athletic practice: before, during, and after training. The results revealed significant gender-based differences in favor of female athletes across all dimensions, and significant differences based on years of sports experience, especially before and during training. No significant differences were observed regarding the type of sport. Given the many external factors influencing sports practice, music can be considered one of the most prominent external stimuli.

Based on the study’s findings, it can be concluded that integrating music into all stages of sports practice may enhance athletes’ motivation, psychological strength, and physical performance. Considering that female athletes and those with more years of experience seem to benefit more, developing personalized music playlists could be a strategic tool for improving performance and well-being. Future studies should be designed with broader and culturally diverse samples and should include various age groups and sport branches. Additionally, tailoring music choices to match exercise intensity and athletes’ emotional states may support a more systematic and effective integration of music into sports performance strategies.

## Data Availability

The raw data supporting the conclusions of this article will be made available by the authors, without undue reservation.

## References

[ref1] Akkuş Ü. (2007). Müziğin insan sağlığı üzerindeki yeri ve önemi. Sosyal Bilimler Araştırmaları Dergisi 2, 98–103.

[ref2] Arıkan A. N. Akoğuz-Yazıcı N. (2022). “Sporcuların bağlılık düzeylerinin incelenmesi: Takım sporları üzerine kesitsel bir analiz” in Akdeniz Spor Bilimleri Dergisi, Akademik Spor Araştırmaları Özel Sayısı, 738–748.

[ref3] Bayraktar B. Kurtoğlu M. (2004). Sporda performans ve performans artırma yöntemleri. Doping ve futbolda performans artırma yöntemleri. İstanbul, 269–296.

[ref4] Bektaş M. Demir O. (2022). Mücadele sporları ile ilgilenen bireylerde müziğin psikolojik sağlamlık, performans ve motivasyon üzerine etkisi. Gaziantep Üniv. Spor Bilimleri Dergisi 7, 417–428. doi: 10.31680/gaunjss.1164794

[ref5] Bentouati E. Romdhani M. Abid R. Khemila S. Garbarino S. Souissi N. (2023). The combined effects of napping and self-selected motivation music during warming up on cognitive and physical performance of karate athletes. Front. Physiol. 14:1214504. doi: 10.3389/fphys.2023.1214504, PMID: 37520834 PMC10372344

[ref6] Birnbaum L. Boone T. Huschle B. (2009). Cardiovascular responses to music tempo during steady-state exercise. J. Exerc. Physiol. Online 12, 50–57.

[ref7] Çakmakçı G. (2021). Savunma Sporları ile İlgilenen Sporcularda Başarı Motivasyonu. [Yüksek Lisans tezi]: Recep Tayyip Erdoğan Üniversitesi.

[ref8] Chen J. Han J. Su P. Wang M. Shi W. Tang D. (2025). Effects of perceived groove in music on cycling performance and intermuscular coherence between trunk and lower limb muscles. J. Sci. Med. Sport 28, 594–601. doi: 10.1016/j.jsams.2025.01.014, PMID: 40011097

[ref9] Cohen J. (2013). Statistical power analysis for the behavioral sciences: Routledge.

[ref10] Creswell J. W. Creswell J. D. (2017). Research design: Qualitative, quantitative, and mixed methods approaches. United State of America: Sage Publications.

[ref11] Creswell C. Nauta M. H. Hudson J. L. March S. Reardon T. Arendt K.. (2021). Research review: recommendations for reporting on treatment trials for child and adolescent anxiety disorders–an international consensus statement. J. Child Psychol. Psychiatry 62, 255–269. doi: 10.1111/jcpp.13283, PMID: 32683742

[ref12] Dereceli E. Dokuzoğlu G. Dereceli Ç. (2023). Sağlık hizmetleri meslek yüksekokulu öğrencilerinin fiziksel aktivitelerden keyif alma düzeylerinin çeşitli değişkenler açısından değerlendirilmesi. Res. Sport Educ. Sci. 25, 40–44. doi: 10.5152/JPESS.2023.23071

[ref13] Ekiz M. A. Atasoy M. (2021). Sportif uygulamalarda müzik: kırşehir ahi evran üniversitesi beden eğitimi ve spor yüksekokulu öğrencileri üzerine bir araştırma. Akdeniz Spor Bilimleri Dergisi 4, 241–250. doi: 10.38021/asbid.954189

[ref14] Erdal G. (2005). Sporda Performansın Artırılmasında Müziğin Etkisi, Erişim. Available online at: http://www.muzikegitimcileri.net/bilimsel/bildiri/G-Erdal_2.html

[ref15] Ersoy E. Başer N. E. (2010). Probleme Dayali Öğrenme Sürecinin Öğrenci Motivasyonuna Etkisi. Int Period Fort Lang. Literat. Hist Turkish Turkic 5, 336–358.

[ref16] Filiz K. (2002). Sporun tanımlanması ve kapsamının belirlenmesi üzerine bir çalışma. Gazi Üniv. Gazi Eğitim Fakültesi Dergisi 22, 204–211.

[ref17] George D. Mallery P. (2016). IBM SPSS statistics 23 step by step 14th edition.

[ref18] Gravetter F. J. Forzano L. A. B. Rakow T. (2009). Research methods for the behavioral sciences. Belmont, CA: Wadsworth Cengage Learning, 656.

[ref19] Greco F. Quinzi F. Chiodo S. Cerulli C. Tranchita E. Bertollo M.. (2024). The effects of pre-task music on choice visual reaction time in elite taekwondo athletes. J. Sci. Med. Sport 27, 276–280. doi: 10.1016/j.jsams.2024.01.002, PMID: 38245434

[ref20] Henriksen K. Diment G. Hansen J. (2011). Professional philosophy: inside the delivery of sport psychology service at team Denmark. Sport Sci. Rev. 20, 5–21. doi: 10.2478/v10237-011-0043-6

[ref21] HİS. (2021). Herkes İçin Spor Federasyonu. Müzik ve Spor İlişkisi. Available online at: https://his.gov.tr/spor-ve-muzik-iliskisi adresinden alındı. (Accessed June 1, 2025).

[ref22] İnal A. N. (2000). Beden eğitimi ve spor bilimlerine giriş. Konya: Desen Ofset Matbaacılık.

[ref23] Jing L. Xudong W. (2008). Evaluation on the effects of relaxing music on the recovery from aerobic exercise-induced fatigue. J. Sports Med. Phys. Fitness 48:102. 18212717

[ref24] Karageorghis C. I. Priest D. L. (2012). Music in the exercise domain: a review and synthesis (part I). Int. Rev. Sport Exerc. Psychol. 5, 44–66. doi: 10.1080/1750984X.2011.631027 22577472 PMC3339578

[ref25] Karayol M. Turhan M. O. (2020). Impact of music in sportive activities scale (IMSAS): validity and reliability assessment. Afr. Educ. Res. J. 8, 297–304. doi: 10.30918/AERJ.82.20.071

[ref26] Kelly P. Williamson C. Niven A. G. Hunter R. Mutrie N. Richards J. (2018). Walking on sunshine: scoping review of the evidence for walking and mental health. Br. J. Sports Med. 52, 800–806. doi: 10.1136/bjsports-2017-098827, PMID: 29858467

[ref27] Koç H. Koç Ö. (2023). Sportif uygulamalarda müzik ve spora bağlılık: Spor merkezi üyeleri üzerine bir araştırma. Ulusal Spor Bilimleri Dergisi 7, 65–81. doi: 10.30769/usbd.1340827

[ref28] Konter E. (1996) Profesyonel futbolcuların bazı kişisel değişkenlerinin durumluk kaygı üzerine etkileri ve durumluk kaygının takım içi ilişkilerine etkisinin araştırılması (3. lig uygulaması) (Master's thesis, Sağlık Bilimleri Enstitüsü)

[ref29] Küçük H. Durmuşoğlu M. V. (2022). Examination of Turkish and foreign female high school students’ attitudes to physical education and sports lesson. J. Pharm. Negat. Results 13, 819–822. doi: 10.47750/pnr.2022.13.S01.101

[ref30] Kurt G. Karaca S. Karaca R. Akrasia Ve zihinsel sağlik kavramlarinin incelenmesi. Spor Disiplinlerarasi Bilimsel Yaklaşimlar, 5.

[ref31] Kuter M. Ozturk F. (1997). Antrenor ve Sporcu El Kitabi. Bursa: Bursa Gazetecilik ve Yayincilik A.S. Matbaasi, s.17.

[ref32] Marshall C. Rossman G. B. (2014). Designing qualitative research (6th ed.). Thousand Oaks, CA: Sage.

[ref33] Mavi S. (2012). Hızlı tempo müziğin taekwondocular üzerindeki etkileri. Ankara: Ankara Üniversitesi Sağlık Bilimleri Enstitüsü.

[ref34] McCall P.. (2014). How to select the right intensity and repetitions for your clients. Ace. Available online at: https://www.acefitness.org/resources/pros/expert-articles/4922/how-to-select-the-right-intensity-and-repetitions-for-your-clients/articles/4922/how-to-select-the-right-intensity-and-repetitions-for-your-clients/adresindenalındı (Accessed June 1, 2025).

[ref35] Messaoudi H. Ouergui I. Delleli S. Ballmann C. G. Ardigò L. P. Chtourou H. (2024). Acute effects of plyometric-based conditioning activity and warm-up music stimuli on physical performance and affective state in male taekwondo athletes. Front. Sports Active Living 5:1335794. doi: 10.3389/fspor.2023.1335794, PMID: 38287965 PMC10822913

[ref36] Miras-Moreno S. Weakley J. Martínez-Zafra L. M. Pérez-Castilla A. (2025). Impact of augmented feedback and music during the bench press resistance exercise: does their combination compromise mechanical performance? Sports Health. 17:19417381251316216. doi: 10.1177/19417381251316216, PMID: 39924649 PMC11808692

[ref37] Ntoumanis N. Biddle S. J. (2000). Relationship of intensity and direction of competitive anxiety with coping strategies. Sport Psychol. 14, 360–371. doi: 10.1123/tsp.14.4.360

[ref38] Ooishi Y. Mukai H. Watanabe K. Kawato S. Kashino M. (2017). Increase in salivary oxytocin and decrease in salivary cortisol after listening to relaxing slow-tempo and exciting fast-tempo music. PLoS One 12:e0189075. doi: 10.1371/journal.pone.0189075, PMID: 29211795 PMC5718605

[ref39] Özen G. (2022). Spor Ve Bilim. İstanbul: Efe Yayıncılık.

[ref40] Penedo F. J. Dahn J. R. (2005). Exercise and well-being: a review of mental and physical health benefits associated with physical activity. Curr. Opin. Psychiatry 18, 189–193. doi: 10.1097/00001504-200503000-00013, PMID: 16639173

[ref41] Petersen J. M. Drummond M. Crossman S. Elliott S. Drummond C. Prichard I. (2023). Mental health promotion in youth sporting clubs: predictors of stakeholder participation. BMC Public Health 23, 1–8. doi: 10.1186/s12889-023-15377-5, PMID: 36915086 PMC10009942

[ref42] Şahin T. Güçlü M. (2019). Sporda psikolojik dayanıklılık. Ankara: Pegem Akademi.

[ref43] Say A. (1998). Müzik tarihi. Ankara: Müzik Ansiklopedisi Yayınları.

[ref44] Sener S. Cantürk E. Topal E. E. (2024). The effects of classical music on pain and spinal mobility during stretching exercises in healthy individuals. Istanbul Gelisim University Journal of Health Sciences. 23, 613–628.

[ref45] Stevens M. J. Lane A. M. (2001). Mood-regulating strategies used by athletes, athletic inside. J. Sport Pychol. 3, 3–10.

[ref46] Terry P. C. Karageorghis C. I. Curran M. L. Martin O. V. Parsons-Smith R. L. (2020). Effects of music in exercise and sport: a meta-analytic review. Psychol. Bull. 146, 91–117. doi: 10.1037/bul0000216, PMID: 31804098

[ref47] Turhan M. Ö. (2021). Sportif uygulamalarda müziğin etkisi ile spora özgü başarı motivasyonu arasındaki ilişkinin incelenmesi. Muş: Muş Alparslan Üniversitesi Sosyal Bilimler Enstitüsü.

[ref48] Turhan M. Ö. Karayol M. (2022). Examination of the relationship between music and success motivation in sports. Int. J. Eurasian Educ. Cult. 7, 1171–1201. doi: 10.35826/ijoecc.577

[ref49] Üngür G. (2021). Zihinsel sağlik ve egzersiz stratejileri. holistik sağlik ve egzersiz, 95.

[ref50] Vatansever Ş. Şahin Ş. Akalp K. Şentürk F. C. (2018). Müziğin maksimal koşu performansına ve egzersiz sonrası toparlanma hızına etkisi. Türkiye Spor Bilimleri Dergisi 2, 61–66. doi: 10.32706/tusbid.486188

[ref51] Vural M. Özdemir A. Şık A. (2019). Karate sporcularinin müzikten etkilenme ve akademik öz yeterliklerinin bazi demografik değişkenlere göre incelenmesi. Beden Eğitimi Spor Bilimleri Dergisi 21, 79–84.

